# YAP1 expression is associated with survival and immunosuppression in small cell lung cancer

**DOI:** 10.1038/s41419-023-06053-y

**Published:** 2023-09-26

**Authors:** Peixin Chen, Chenglong Sun, Hao Wang, Wencheng Zhao, Yan Wu, Haoyue Guo, Caicun Zhou, Yayi He

**Affiliations:** 1 Department of Medical Oncology, Shanghai Pulmonary Hospital, Tongji University Medical School Cancer Institute, Tongji University School of Medicine, No 507 Zhengmin Road, Shanghai, 200433 People’s Republic of China; 2 Tongji University, No 1239 Siping Road, Shanghai, 200433 People’s Republic of China; 3 Radiotherapy Department, Anhui No. 2 Provincial People’s Hospital, Hefei, 230041 Anhui People’s Republic of China

**Keywords:** Tumour heterogeneity, Cancer microenvironment, Targeted therapies, Small-cell lung cancer

## Abstract

Immunotherapy is considered a major breakthrough in the treatment of small cell lung cancer (SCLC), although its anti-tumor efficacy is limited. With a high degree of malignancy and high heterogeneity, SCLC is difficult to treat in the clinic. A new combination strategy is urgently needed to further improve the efficacy of immunotherapy in patients with SCLC. By immunofluorescence, 100 SCLC patients in a local cohort were classified into the SCLC-A (high ASCL1 expression; *n* = 36), SCLC-N (high NEUROD1 expression; *n* = 32), SCLC-P (high POU2F3 expression; *n* = 14), and SCLC-Y (high YAP1 expression; *n* = 18) subtypes. Each SCLC molecular subtype represented different prognoses, tumor microenvironment traits, and immunotherapy sensitivities. Analysis of both the local and public cohorts suggested that the SCLC-Y subtype exhibited the worst clinical outcome (*p* < 0.05) when compared with other subtypes. SCLC with high YAP1 expression was characterized by high PD-L1 expression, high stromal score, T-cell functional impairment, and a close relationship with immune-related pathways. YAP1 upregulated PD-L1 expression and suppressed T cell activation, thus leading to immune evasion. In in vitro experiments, blockade of YAP1 promoted cancer cell apoptosis, immune cell proliferation, T-cell activation, and cytotoxic T-cell infiltration, thus further potentiating the efficacy of immunotherapy in patients with the SCLC-Y subtype.

## Introduction

Small cell lung cancer (SCLC), characterized by a high degree of malignancy and rapid infiltrating tumor growth, accounts for approximately 10–15% of the total population of lung cancer cases [1–3]. More than half of SCLC patients are initially diagnosed at an advanced stage and are thus not candidates for surgical therapy [4, 5]. Patients with unresectable SCLC have a dismal survival prognosis, with a 5-year survival rate of only 2–6% [4, 5]. According to the standard treatment guidelines for SCLC, a platinum-based regimen (cisplatin or carboplatin plus etoposide) was recommended for a few decades [6–8]. Recently, an immune checkpoint (IC) inhibitor, atezolizumab, was added to the list of first-line therapies recommended for patients with advanced SCLC [9, 10]. Other IC inhibitors, such as durvalumab and serplulimab, also significantly improved the prognosis of patients with SCLC [11, 12]. Clinical trials suggested that immunotherapy plus chemotherapy conferred a clinical benefit of a nearly 2–4.5 months increase in survival time compared with chemotherapy alone [9, 11–13]. However, the proportion of SCLC patients who experienced relapse within a year after receiving immunotherapy was as high as 85% [9, 10, 13]. A major challenge related to SCLC is how to improve the clinical efficacy of its treatment. In addition, no survival benefit of nivolumab was observed in patients with chemo-resistant SCLC [14]. Clearly, there is a need to identify SCLC patients who might be sensitive to immunotherapy and achieve satisfactorily prolonged survival. The development of more effective combined therapies or targeted drugs is also worthwhile.

In recent years, Rudin C et al. reviewed the existing SCLC subtype classification and proposed novel molecular subtypes of SCLC, including the SCLC-A subtype, the SCLC-N subtype, the SCLC-Y subtype, and the SCLC-P subtype [15]. The new SCLC molecular subtypes are defined by the relative expression levels of four key transcription factors: achaete-scute homologue 1 (ASCL1), neurogenic differentiation factor 1 (NEUROD1), yes-associated protein 1 (YAP1), and POU class 2 homeobox 3 (POU2F3) [15]. Rudin C et al. found that ASCL1, NEUROD1, YAP1, and POU2F3 exhibited the highest relative expression levels in the SCLC-A, SCLC-N, SCLC-Y, and SCLC-P subtypes, respectively [15]. Moreover, the SCLC-A and SCLC-N subtypes were classified as neuroendocrine subtypes with high INSM transcriptional repressor 1 (INSM1) expression. The non-neuroendocrine subtypes were the SCLC-Y and SCLC-P subtypes. This classification scheme was conducive to the identification of subtype-specific drug sensitivities and therapeutic targets. Specifically, delta-like canonical Notch ligand 3 (DLL3), BCL2 apoptosis regulator (BCL2), and lysine demethylase 1 A (LSD1) were considered promising targets in SCLC-A tumors. Rudin C et al. also noted that SCLC-N tumors but not unselected SCLC tumors might demonstrate a unique vulnerability to the oncolytic virus SVV. ASCL1, one of the neuronal transcription factors, is considered a crucial oncogene in SCLC [16]. NEUROD1, another important neuronal regulator, regulates biological processes in various types of cancer cells, such as SCLC, medulloblastoma, and colorectal cancer cells [17–20]. In the Hippo signaling pathway, YAP1, along with TAZ, activates TEADs, thus affecting cell proliferation, organ size, drug sensitivity, and the tumor microenvironment (TME) [21–23]. In SCLC, the existing studies of POU2F3 have mainly focused on its function in chemosensory cells [24]. In melanoma, the expression level of POU2F3 was found to be downregulated by microRNA-27a, resulting in the modulation of cell proliferation and metastasis [25].

As critical regulators of the immune system, ICs play important roles in the processes related to tumor development, tumor progression, and drug-resistance [26]. Overexpression of ICs might contribute to immune escape in various human tumors [27]. Several ICs, such as programmed death-1 (PD-1), programmed death ligand-1 (PD-L1), T cell immunoglobulin and mucin-domain containing-3 (TIM-3), galectin-9, major histocompatibility complex Class II (MHC II), lymphocyte activation gene-3 (LAG-3), OX40, OX40 ligand (OX40L), and CD39, have been considered as potential targets of immunotherapy [28]. Interactions of PD-1 and PD-L1 suppressed T-cell functions and initiated the programmed death of immune cells, thus leading to immune escape [29]. The clinical application of PD-1/PD-L1 inhibitors dramatically prolonged the survival of lung cancer patients [30]. The expression levels of TIM-3 and galectin-9 (a TIM-3 ligand) on CD4+ and CD8 + T cells were up-regulated. Activation of TIM-3/galectin-9 signaling inhibited immune cell activation and increased the regulatory T-cell (Treg) abundance [31]. LAG-3 was found to be selectively expressed on activated T cells, natural killer cells, and dendritic cells. Along with its ligand MHC II, LAG-3 induces T-cell dysfunction and death [32, 33]. High expression of OX40 and OX40L was found on cytotoxic T cells and Tregs. In the TME, overexpression of OX40 and OX40L was correlated with immune activation and anti-tumor effects [34, 35]. However, in patients with early-stage NSCLC, liver cancer, and hematological malignancies, high OX40/OX40L expression indicated a poor prognosis [36–38]. CD39 can exert immunosuppressive effects by promoting adenosine production [39, 40]. The efficacy of a CD39 inhibitor (IPH5201) in combination with a PD-1/PD-L1 inhibitor (pembrolizumab/durvalumab) in advanced solid tumors was evaluated in clinical trials [40]. Currently, several IC inhibitors and bispecific antibodies have been developed and have been evaluated in clinical trials [26, 28, 32, 41]. Comprehensive studies of the expression patterns of the above ICs in the four SCLC molecular subtypes might facilitate the development of new subtype-specific combination treatment schemes. In this article, by immunohistochemical (IHC) and immunofluorescence (IF) staining, we measured the protein expression levels of ASCL1, NEUROD1, YAP1, and POU2F3 and their unique co-expression patterns with ICs in SCLC. We investigated the immune landscape of the defined SCLC molecular subtypes. Then, we evaluated the correlations between SCLC molecular subtypes and prognosis in a local cohort and a public cohort. In in vitro experiments, the important roles of YAP1 in immunotherapy efficacy, PD-L1 expression, and immune cells proportion and function were investigated. Functional profiling of YAP1 in SCLC was performed in a public dataset.

## Patients and methods

### Patients

A total of 100 SCLC patients were enrolled in the study cohort. Histopathologic classification was conducted by two independent pathologists. We used the Tumor–Node–Metastasis (TNM) Classification of Malignant Tumors, 8th edition. The ethics committee of Shanghai Pulmonary Hospital carefully reviewed the present study and gave permission for its performance. All participants gave informed consent.

### Expression levels of YAP1, ASCL1, NEUROD1, POU2F3, and other immune markers

For IF staining, first, 4 µm sections of formalin-fixed, paraffin-embedded tissues were dewaxed with xylene. Then, the antigen retrieval process was performed by the high-pressure method. In order to reduce nonspecific binding, we used bovine serum albumin (G5001, Servicebio). Later, lung slides were incubated with a primary antibody in accordance with the manufacturer’s instructions. After rinsing with phosphate-buffered saline (G4202, Servicebio), the sections were incubated with fluorophore-conjugated secondary antibodies for one hour in a dark room. Finally, the DAPI reagent (G1012, Servicebio) was applied for nuclear counterstaining. The primary antibodies used in the study included anti-YAP (sc-101199, Santa Cruz Biotechnology), anti-ASCL1 (sc-390794, Santa Cruz Biotechnology), anti-NEUROD1 (ab205300, Abcam), and anti-POU2F3 (bs-21046R, Bioss). The fluorophore-conjugated secondary antibodies used for double IF staining included Alexa Flour 488-conjugated goat anti-rabbit IgG (GB25303, Servicebio) and Cy3-conjugated goat anti-mouse IgG (GB21301, Servicebio).

The expression levels of CD3, CD4, CD8, forkhead box protein P3 (FOXP3), PD-1, PD-L1, TIM-3, MHC II, LAG-3, galectin-9, OX40, OX40L, and CD39 were measured by IHC analysis. The detailed IHC analysis procedures and the primary and secondary antibodies used for IHC staining were reported in our published articles [42, 43].

Images were captured under an inverted microscope (IX73, Olympus). The protein expression levels of ASCL1, NEUROD1, YAP1, POU2F3, CD3, CD4, CD8, FOXP3, PD-1, PD-L1, TIM-3, MHC II, LAG-3, galectin-9, OX40, OX40L, and CD39 were quantified in each field of view at 20x magnification. Three random fields of view were selected per slide. The expression levels of ICs on tumor cells and tumor-infiltrating lymphocytes (TILs) were independently reviewed and assessed by two certified pathologists. TILs were defined as lymphocytes infiltrating within the tumor region. With the assistance of two pathologists, the percentages of CD3+ immune cells, CD4+ immune cells, CD8+ immune cells, and FOXP3+ immune cells in the tumor tissues were determined (0 to 100%). The average scores determined by the two pathologists were designated as the staining results.

### Molecular subtype identification by the NMF method

Non-negative matrix factorization (NMF) algorithm is an important method in unsupervised clustering analysis. For the identification of SCLC molecular subtypes, a highly robust NMF algorithm was used to analyze the SCLC cohort. Clinical SCLC patients with similar expression characteristics were classified into the same cluster. The NMF R package was installed and used for clinical cluster identification [44]. A cophenetic coefficient plot was used to determine the optimal cluster number. A resampling algorithm was adopted to decrease the probability of random error and increase the stability of the cluster analysis. The final classification results were acquired by bootstrapping with 1000 resamples.

### Cell culture

DMS114, H2286, SHP77, H446, and H526 cells were obtained from ATCC. All cell lines were negative for mycoplasma contamination. All cells were cultured in RPMI 1640 medium supplemented with 10% FBS and 1% penicillin/streptomycin. Cells were grown in an incubator at 37 °C in 5% CO_2_. The expression profiles of the above SCLC cell lines were extracted from the CellMinerCDB database (https://discover.nci.nih.gov/cellminercdb/), as previously provided in detail [45].

### Establishment of YAP1-overexpression cells

To generate SHP77, H446, and H526 cell lines with YAP1 overexpression, the YAP1 overexpression lentivirus (CMV enhancer-MCS-3FLAG-YAP1-ZsGreen1-T2A-puromycin, 70186-1, Shanghai GeneChem Co.,Ltd.) was applied according to the manufacturer’s instructions. The negative control virus (CON522, Shanghai GeneChem Co.,Ltd.) was used as a negative control. Stably transduced SCLC cells were selected by incubation with puromycin (2.5 µg/ml, 631305, Clontech) for 48 h. The transduction efficiency was assessed by fluorescence microscopy and quantitative RT‐PCR. Then, the YAP1-overexpressing SCLC cells were expanded for subsequent experiments.

### Cell viability assay

Peripheral blood mononuclear cells (PBMCs) were isolated by gradient centrifugation. SCLC cells and PBMCs were cocultured in 96-well plates at a 20:1 effector-to-target ratio. After a 24-hour incubation at 37 °C in 5% CO_2_, drug-free medium was replaced with drug-containing medium. To establish the etoposide and cisplatin (EC) group, SCLC cells were treated with medium containing 0.25 µM etoposide (S1225, Selleck Chemicals) and 0.5 µM cisplatin (S1166, Selleck Chemicals). To establish the chemoimmunotherapy group, SCLC cells were cultured in medium containing 0.25 µM etoposide, 0.5 µM cisplatin, and 10 µg/mL PD-L1 inhibitor (A2004, Selleck Chemicals; atezolizumab). To establish the immunotherapy group, SCLC cells were cultured in medium containing 10 µg/mL atezolizumab. To establish the YAP1 inhibitor plus chemoimmunotherapy group, SCLC cells were cultured in medium containing 2 µM verteporfin (S1786, Selleck Chemicals), 10 µg/mL atezolizumab, 0.25 µM etoposide, and 0.5 µM cisplatin. To establish the control experiment, SCLC cells were treated with fresh medium containing an equivalent volume of PBS. After 5 days, cell viability was measured with Cell Counting Kit-8 (CCK8) reagent (Dojindo). The absorbance at 450 nm was recorded after a 3-hour incubation with CCK8 solution. For each condition, three replicates were analyzed. The cell survival rate of cells was calculated by normalization to the control wells without drug exposure.

### Colony formation assay

Two thousand cells per well were plated in six‐well culture plates. After incubation for 24 h, DMS114 and H2286 cells were co-cultured with PBMCs in drug-containing medium for 48 h. To establish the chemoimmunotherapy group, 10 µg/mL atezolizumab, 0.25 µM etoposide, and 0.5 µM cisplatin were added to the culture medium. To establish the YAP1 inhibitor plus chemoimmunotherapy group, SCLC cells were treated with 2 µM verteporfin, 10 µg/mL atezolizumab, 0.25 µM etoposide, and 0.5 µM cisplatin. Then, the drug-containing medium was replaced with fresh medium. After two weeks, colonies were washed once with PBS buffer, fixed with 4% paraformaldehyde for 30 minutes, and then washed thoroughly with PBS buffer. Next, the colonies were stained with crystal violet (1 mL) for 20 minutes. Finally, colonies of at least 30 cells were counted under a microscope.

### Flow cytometry

Flow cytometry was applied to evaluate the apoptosis rate and stemness of cancer cells, and the changes in immune cell infiltration and function after drug exposure. The effects of YAP1 on PD-L1 expression and immune cells were also assessed by flow cytometry. According to the manufacturer’s guidelines, cells were collected and first stained using Fixable Viability Dye-EF506 (65-0866-14, Thermo Fisher Scientific) to distinguish between live and dead cells. Subsequently, the cells were stained with antibodies specific for surface markers for 15 minutes at room temperature. The surface antibodies included anti-human C45-PerCP (304026, Biolegend), CD3-BV570 (300436, Biolegend), CD4-BV750 (747202, BD Pharmingen), CD8-APC/Fire 810 (344764, Biolegend), PD-L1-BV650 (563740, BD Pharmingen), Fas-SB600 (63-0959-42, Thermo Fisher Scientific), and HLA-DR-PE-CY7 (560651, BD Pharmingen) antibodies. Then, the cells were washed with 1x Annexin V Binding Buffer (51-66121E, BD Pharmingen) and stained with Annexin V-FITC (51-65874X, BD Pharmingen) antibody for 30 minutes. Finally, cells were processed using the fixation/permeabilization solution (00-5521-00, Thermo Fisher Scientific) and further stained with anti-YAP1-PE (14710 S, Cell Signaling Technology), anti-Ki67-PerCP-Cy5.5 (350520, Biolegend), and anti-Granzyme-B-AF700 (372222, Biolegend) antibodies for 30 minutes.

To assess the stemness of tumor cells after drug treatment, two stemness markers, CD133 and CD44, were evaluated. DMS114 and H2286 cells were cultured in medium-containing drugs (atezolizumab plus etoposide and cisplatin; verteporfin plus atezolizumab, etoposide and cisplatin; or an equivalent volume of PBS) for 5 days. After preparation of single-cell suspensions, SCLC cells were stained with live/dead stain (65-0866-14, Thermo Fisher Scientific). Then, surface markers on the cells were stained with anti-CD45-PerCP (304026, Biolegend), anti-CD133-APC (566596, BD Pharmingen), and anti-CD44-PE (550989, BD Pharmingen) antibodies for 30 minutes at 4 °C.

All flow cytometric analyses were performed on a Northern Lights-CLC spectral flow cytometer (Cytek Biosciences, Inc). All flow cytometry data were analyzed by FlowJo software (version 10.8.1).

### Selection of external validation SCLC datasets

In order to further verify the critical roles of the molecular subtype and YAP1 in SCLC, we applied the Gene Expression Omnibus (GEO) database (https://www.ncbi.nlm.nih.gov/geo/) and the cBioPortal database (https://www.cbioportal.org). Both GEO and cBioPortal are genome databases that contain high-throughput sequencing data from multiple studies. The GEO or cBioPortal datasets used in our study were required to adhere to inclusion criteria regarding several aspects: mRNA sequencing data for human SCLC samples, full results of mRNA sequencing analysis, and survival data of SCLC patients. The established exclusion criteria include repetitive datasets, datasets with incomplete gene expression data, and datasets of nonprotein-coding gene sequencing results. Finally, 49 samples from the GSE60052 dataset [46] and 77 patients from the cBioPortal dataset [47] were determined to meet the inclusion criteria. With the addition of 6 SCLC patients extracted from our center [48, 49], the public cohort was composed of a total of 132 SCLC patients with prognostic data.

### Analysis of the YAP1 signaling pathway in SCLC

After data selection and preprocessing, we confirmed the differentially expressed genes (DEGs) between the high YAP1 group and the low YAP1 group with the R package limma. The p value of each DEG was less than 0.05. The results of signaling pathway analysis of the DEGs were visualized with the clusterProfiler R package. The bar graph of the Gene Ontology (GO) analysis results showed the enrichment of DEGs in cellular component, biological process, and molecular function terms. The dot plot of the Kyoto Encyclopedia of Genes and Genomes (KEGG) pathway analysis results highlighted the complex molecular interaction networks of the DEGs. Detailed annotations of the GO (http://www.geneontology.org/) and KEGG analysis results (https://www.kegg.jp/kegg/) are available online. After adjusting the p values by a statistical method, we obtained the corresponding q values. A q value of less than 0.01 was considered to indicate a statistically significant difference.

Independent of the p values and fold changes in the expression of genes, the Gene Set Enrichment Analysis (GSEA) was applied to investigate the pathway enrichment of the global gene expression profile [50]. Each parameter in the GSEA software (V.4.0.3) was left at the default setting. For each biological pathway, the enrichment score (ES) and p value are shown in the enrichment plot. After the selection of meaningful GSEA gene sets by considering the ES and false discovery rate (FDR), gene overlap analysis and leading edge analysis were carried out.

### The landscape of the TME in SCLC

In order to explore the TME landscape in SCLC, the Estimation of STromal and Immune cells in MAlignant Tumours using Expression data (ESTIMATE) algorithm and CIBERSORTx suite were used. First, by means of the ESTIMATE R package, the immune score, stromal score, and tumor purity of each SCLC sample were calculated [51]. On the basis of the gene expression data, the immune score and the stromal score represented the infiltration of immune cells and stromal cells, respectively. Later, the convenient CIBERSORTx tool (https://cibersortx.stanford.edu/) was used to further quantify the abundances of 22 kinds of immune cells in clinical SCLC tissues [52]. A machine learning method, linear support vector regression, was innovatively used in the CIBERSORTx algorithm [52, 53]. In addition, the CIBERSORTx algorithm showed an outstanding advantage in deep deconvolution.

### Statistical analysis

Correlation coefficients between the expression levels of subtype markers and other biomarkers were determined by Pearson correlation analysis. A t test was applied for comparisons differences between two groups, while the Kruskal‒Wallis test was used for comparisons among three or four groups. Kaplan–Meier survival analysis with the log-rank test was conducted to identify prognostic differences between two groups or among more than two groups. The corresponding 95% confidence intervals (95% CIs) were calculated. For all comparisons, statistical significance was assumed at a p value of < 0.05. For all processes in data processing and visualization, we used two free software programs: SPSS software (V.22.0) and R Programming Language for Windows (V.4.0.1).

## Results

### Identification of SCLC subtypes in the primary cohort

The clinical information of the 100 patients and pathological characterization of SCLC tissues in the primary cohort are summarized in Table S1. The patients ranged in age from 38 to 81 years (median 63.5 years). Most participants were men (82/100), while only 18.0% (18/100) were women. In addition, most patients had stage I or II SCLC (60/100).

**Fig. 1:**
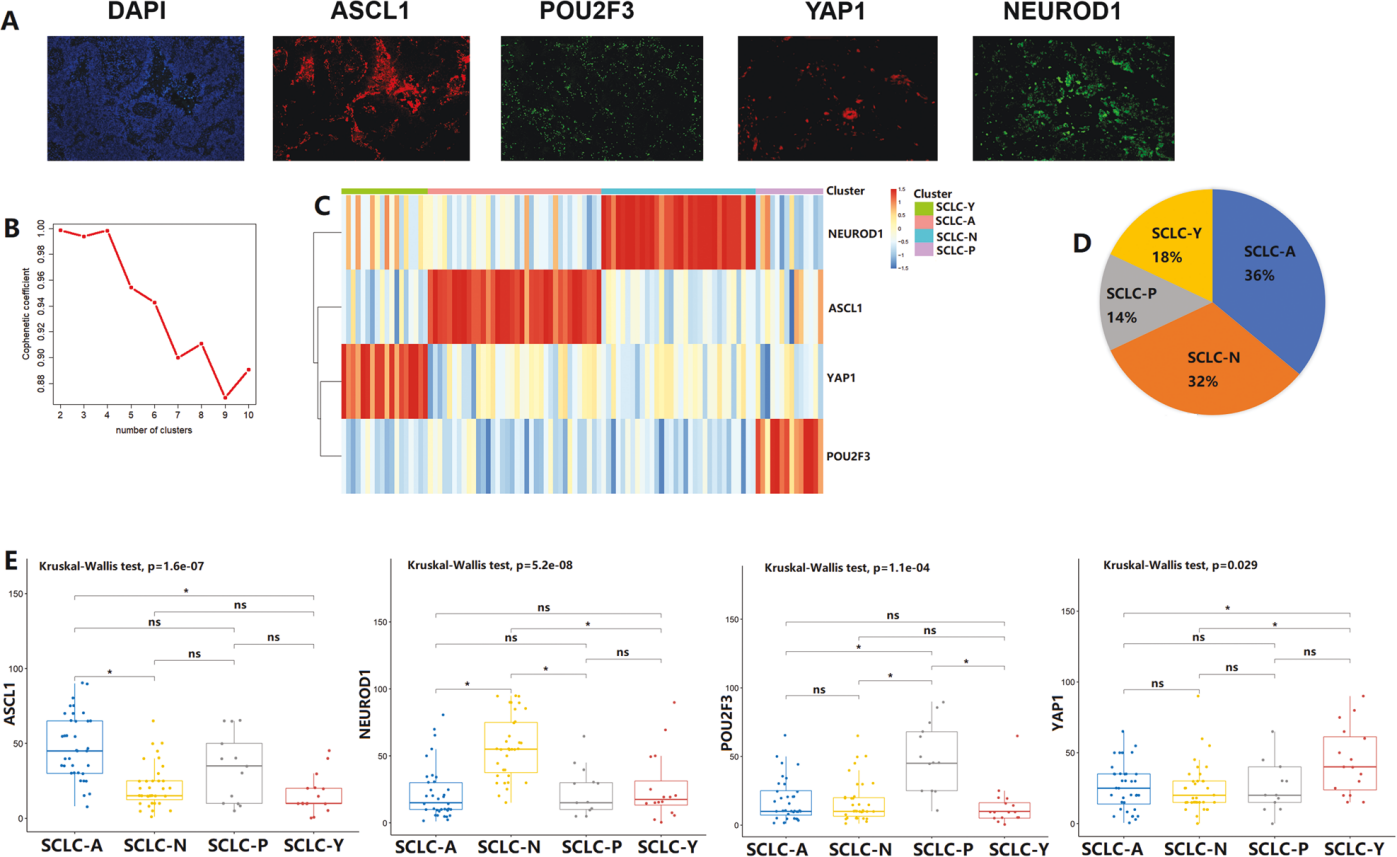
Small cell lung cancer (SCLC) molecular subtypes identification by the non-negative matrix factorization (NMF) algorithm. **A** The protein expression levels of achaete-scute homologue 1 (ASCL1), neurogenic differentiation factor 1 (NEUROD1), yes-associated protein 1 (YAP1), and POU class 2 homeobox 3 (POU2F3) in SCLC tumors. **B** Cophenetic correlation from NMF analysis of 100 SCLC samples. **C** NMF consensus matrix of 4 SCLC molecular subtypes. **D** The proportion of 4 SCLC molecular subtypes in the primary cohort. **E** The expression levels of ASCL1, NEUROD1, POU2F3, and YAP1 among 4 SCLC subtypes. ns, not significant; *p < 0.05.

Expression data for the four key transcription regulators were available for a total of 100 SCLC samples (Fig. 1A). Based on the NMF algorithm, four clusters with the highest cophenetic coefficient values were identified (Fig. 1B). On the basis of the expression phenotypes of the four markers, we divided these samples into four molecular subtypes: SCLC-A subtype (ASCL1-high), SCLC-N subtype (NEUROD1-high), SCLC-Y subtype (YAP1-high), and SCLC-P subtype (POU2F3-high). More than half of the clinical cases were classified as the SCLC-A subtype (36/100) or the SCLC-N subtype (32/100). The SCLC-Y subtype and the SCLC-P subtype accounted for 18% (18/100) and 14% (14/100) of the cases, respectively (Fig. 1C, D). We further evaluated the expression differences in the four key transcriptional regulators among the four SCLC subtypes (Fig. 1E). ASCL1 (Kruskal-Wallis test, p = 1.6e-07), NEUROD1 (Kruskal-Wallis test, p = 5.2e-08), POU2FE (Kruskal-Wallis test, p = 1.1e-04), and YAP1 (Kruskal-Wallis test, p = 0.029) were differentially expressed among the four groups. The SCLC-A group showed the highest ASCL1 expression level. Similarly, the highest expression levels of NEUROD1, POU2FE3, and YAP1 were observed in the SCLC-N subtype group, the SCLC-P subtype group, and the SCLC-Y subtype group, respectively.

### Immune landscapes of the four SCLC subtypes

**Fig. 2:**
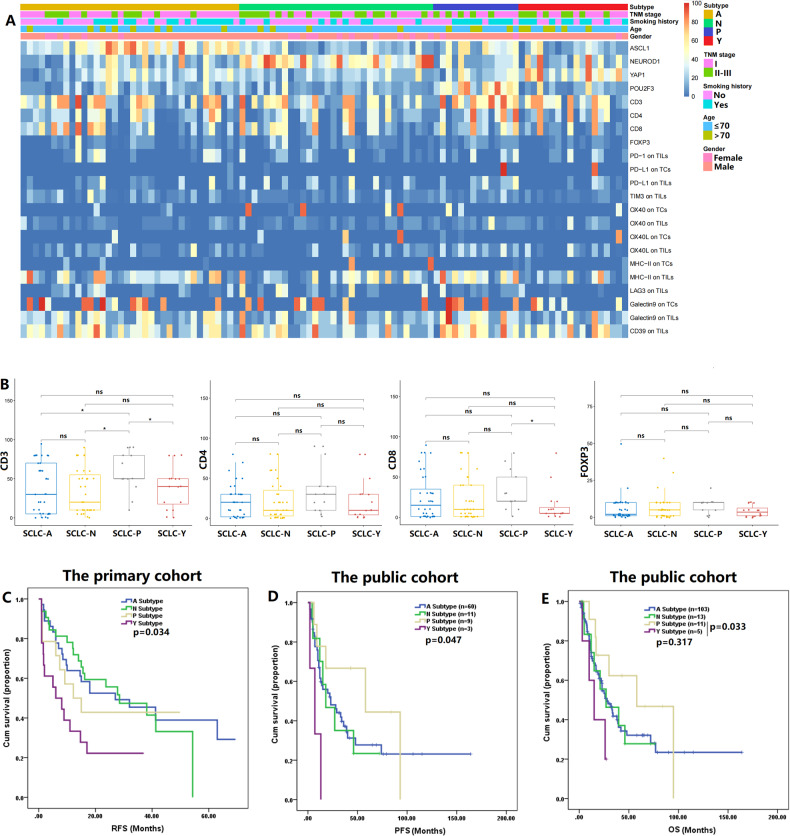
The immune landscape and prognosis of four small cell lung cancer (SCLC) molecular subtypes. **A** The heatmap of four key transcriptional regulators and other immune markers in the primary cohort. Apart from SCLC molecular subtype, other clinical features were also annotated, including age, gender, smoking history, and TNM stage. **B** Differences in immune cell infiltration across the four SCLC subtypes. **C, D, E** Survival analysis by SCLC molecular subtypes in the primary and public cohorts. ASCL1 achaete-scute homologue 1, FOXP3 forkhead box protein P3, LAG3 lymphocyte activation gene-3, MHC-II major histocompatibility complex Class II, NEUROD1, neurogenic differentiation factor 1, ns not significant, OS overall survival; OX40L OX40 ligand, PFS progression-free survival, POU2F3 POU class 2 homeobox 3, PD-1 program death-1, PD-L1 program death-ligand 1, RFS recurrence-free survival, TCs tumor cells, TILs tumor infiltrating lymphocytes, TIM3 T cell immunoglobulin and mucin-domain containing-3, YAP1 yes-associated protein 1; *p < 0.05.

Fig. 2A depicts the expression profiles of ASCL1, NEUROD1, YAP1, POU2F3, and other immune markers in the primary cohort. We further compared immune cell infiltration and IC expression patterns across the four subtypes (Fig. 2B and Figures S1, S2). The proportion of CD3+ immune cells was the highest in the SCLC-P subtype group (both *p* < 0.05), followed by the SCLC-Y subtype group. The SCLC-P subtype group also exhibited significantly higher expression levels of CD8 than the SCLC-Y subtype group (*p* = 0.01). SCLC-P subtype tumors showed higher expression levels of CD4 and FOXP3 than tumors of the other subtypes. However, no statistically significant differences were found in CD4 and FOXP3 expression across the subtypes. In addition, the expression levels of CD3 and CD4 were higher in the SCLC-P/Y (SCLC-P and SCLC-Y) group (*n* = 32) than in the SCLC-A/N (SCLC-A and SCLC-N) group (*n* = 68, Figure S1A). The SCLC-P subtype group showed high expression levels of TIM3, OX40, OX40L, galectin9 and MHC-II on TILs, indicating that patients with the SCLC-P subtype might benefit from treatment with TIM3, OX40, OX40L, galectin9 or MHC-II inhibitors (Figure S2). In order to comprehensively explore the co-expression patterns of the four key transcriptional markers, immune markers, and ICs, we performed correlation analysis. The correlation matrices illustrated that there were moderate correlations among the above markers (Figure S1B). The expression of ASCL1 was significantly related to galectin9 expression on TCs (*r* = 0.206, *p* = 0.04). A similar association was also found for NEUROD1 expression and MHC-II expression on TCs (*r* = 0.203, *p* = 0.042). These results suggested that the SCLC-A subtype with high ASCL1, might be sensitive to galectin9 inhibitors. There was a significant correlation between NEUROD1 expression and OX40 expression on TCs (*r* = 0.238, *p* = 0.017). These preliminary identified positive correlations supported the idea that OX40 might be a key target in the SCLC-N subtype (high NEUROD1 expression). More preclinical experiments and prospective clinical trials are required to verify the above hypothesized subtype-specific vulnerabilities based on this exploratory clinical analysis.

### Prognostic differences across the four SCLC subtypes

After SCLC molecular subtype recognition in the primary cohort, survival analysis was applied to reveal prognostic differences among the four subtype groups (Fig. 2C). On the whole, there were significant differences in RFS among the four subtypes (*p* = 0.034, Fig. 2C). The clinical outcome was the least favorable in the SCLC-Y subtype group (RFS: SCLC-Y subtype, 12.7 months, 95% CI: 6.3–19.0 vs. SCLC-A subtype, 35.0 months, 95% CI: 25.4–44.7 vs. SCLC-N subtype, 30.4 months, 95% CI: 22.9–38.0 vs. SCLC-P subtype, 25.1 months, 95% CI: 13.8–36.5). When compared with the SCLC-A or SCLC-N subtype group, the SCLC-P subtype group exhibited a shorter RFS, but the differences were not statistically significant.

We further compared prognosis among the four SCLC subtypes in the public SCLC cohort (Table S2). Of the 132 SCLC patients with survival data, 78.8% were males (104/132). Slightly over half of the patients had stage I or II disease (56.1%, 74/132). The NMF algorithm was applied to identify SCLC molecular subtypes in the GSE60052 cohort (Figure S3). In the other two public cohorts, we extracted subtype-related data from the published articles. In the total public cohort, the majority of patients were classified into the SCLC-A subtype (78.0%, 103/132). The proportions of patients with the SCLC-N, SCLC-P, and SCLC-Y subtypes were 9.8%, 8.3%, and 3.8%, respectively. The survival analysis revealed that the SCLC-Y subtype group had the shortest PFS and OS times among the subtype groups (PFS: 7.3 months, 95% CI: 1.1–13.6 vs. 31.5–57.9 months, 95% CI: 15.0–85.9, *p* = 0.047; OS: 16.3 months, 95% CI: 8.1–24.5 vs. 36.8–60.5 months 95% CI: 20.8–84.6; Fig. 2D, E). The OS time was significantly shorter in the SCLC-Y subtype group than in the SCLC-P subtype group (*p* = 0.033).

### YAP1 inhibitor potentiates the immunotherapy response in SCLC-Y subtype

Both our survival analysis results and bioinformatics analysis results revealed poor prognosis of patients with the SCLC-Y subtype. A literature search showed that the SCLC-Y subtype might be sensitive to immunotherapy [15]. Moreover, the results of in vitro and in vivo experiments suggested that YAP1 promoted drug resistance, tumor growth, and progression in SCLC [54, 55]. Given these findings, we proposed a hypothesis that the combination of immunotherapy and YAP1 inhibitor might further improve the efficacy of immunotherapy in SCLC-Y subtype.

**Fig. 3:**
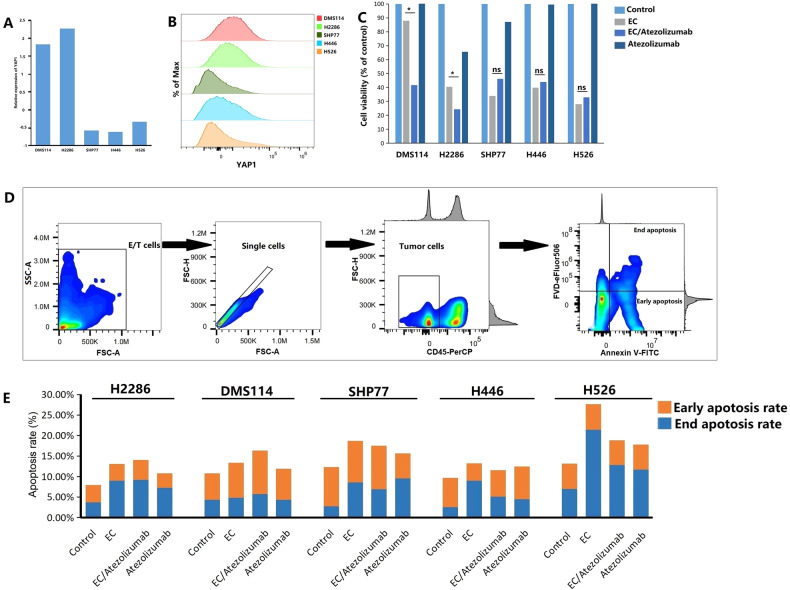
Efficacy of immunotherapy in small cell lung cancer (SCLC) cell lines. **A** mRNA expression levels of YAP1 in different SCLC cell lines. **B** Protein expression level of YAP1 in different SCLC cell lines. Scale bars indicate median fluorescence intensity. **C** Cell viability of SCLC by Cell Counting Kits-8 (CCK8) assay in the control group (equivalent volume of PBS), the EC group (etoposide plus cisplatin), the EC/Atezolizumab group (atezolizumab plus etoposide and cisplatin), and the Atezolizumab group. **D** Gating strategy of early and late apoptotic-tumor cells after drug exposure. **E** The proportions of early and late apoptotic-tumor cells in different groups. EC etoposide plus cisplatin, FVD Fixable Viability Dye, ns not significant, E/T cells effector and target cells, YAP1 yes-associated protein 1; *p < 0.05.

By RNA-sequencing and flow cytometric analyses, we determined that the expression level of YAP1 in SCLC-Y cell lines (DMS114 and H2286) was higher than that in SCLC-A/N/P cell lines (SHP77, H446, and H526; Fig. 3A-B). To validate the promising anti-cancer activity of immunotherapy against the SCLC-Y subtype, we analyzed the viability of SCLC cells by a CCK8 assay. As shown in Fig. 3C, chemoimmunotherapy (atezolizumab plus etoposide and cisplatin) significantly inhibited cell proliferation in SCLC-Y cell lines when compared with chemotherapy alone (etoposide plus cisplatin; both *p* < 0.05). In the SCLC-A/N/P cell lines (SHP77, H446, and H526), the difference in cell viability between the EC plus atezolizumab group and the EC group was not statistically significant (both *p* > 0.05). In vitro, the effects of immunotherapy on apoptosis were quantified by flow cytometric analysis (Fig. 3D, E). The gating strategy used for identifying early and late apoptotic tumor cells after drug exposure is shown in Fig. 3D. In SCLC-Y cell lines, a higher apoptosis rate was found in the EC plus atezolizumab group than in the chemotherapy (etoposide plus cisplatin) group (H2286: 13.07% vs. 14.03%; DMS114: 13.36% vs. 16.33%; Fig. 3E). On the contrary, the addition of PD-L1 inhibitor failed to promote apoptosis in SCLC-A/N/P cell lines (SHP77, H446, and H526) compared with chemotherapy alone (etoposide plus cisplatin; Fig. 3E). Collectively, the consistent results of the CCK-8 assay and flow cytometric analysis verified the important effects of immunotherapy on inhibiting cell proliferation and increasing tumor cell apoptosis in the SCLC-Y subtype, but not in other subtypes.

**Fig. 4:**
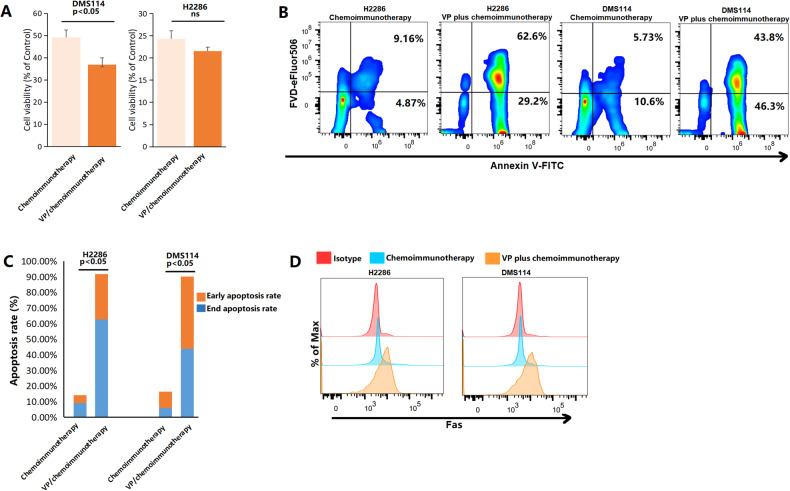
YAP1 inhibitor potentiates immunotherapy in small cell lung cancer (SCLC)-Y subtype. **A** Cell viability of SCLC by Cell Counting Kits-8 (CCK8) assay. **B, C** The proportions of early and late apoptotic-tumor cells in the chemoimmunotherapy (atezolizumab plus etoposide and cisplatin) group and the VP/chemoimmunotherapy (verteporfin plus atezolizumab, etoposide and cisplatin) group. **D** Median fluorescence intensity of Fas in different groups. FVD Fixable Viability Dye, ns not significant, VP Verteporfin.

In the DMS114 cell line, the combination of verteporfin and chemoimmunotherapy showed significant synergistic cytotoxic effect (Fig. 4A, *p* < 0.05). A similar cytotoxic effect of verteporfin plus chemoimmunotherapy (verteporfin plus atezolizumab, etoposide and cisplatin) was found in H2286 cells, another SCLC-Y cell line. In these two SCLC-Y cell lines, dramatically increased proportions of early and late apoptotic cells were found in the verteporfin plus chemoimmunotherapy group compared with the chemoimmunotherapy group (early apoptotic cells: 4.87% vs. 29.2% in H2286; 10.6% vs. 46.3% in DMS114; late apoptotic cells: 9.16% vs. 62.6% in H2286; 5.73% vs. 43.8% in DMS114; Fig. 4B). When compared with chemoimmunotherapy alone (atezolizumab plus etoposide and cisplatin), the addition of verteporfin significantly increased the total proportions of apoptotic SCLC-Y tumor cells (H2286: 14.03% vs. 91.8%; DMS114: 16.33% vs. 90.10%; Fig. 4C, both *p* < 0.05). The median fluorescence intensity (MFI) of Fas, an indicator of apoptosis, was higher in the YAP1 inhibitor plus chemoimmunotherapy group (verteporfin plus atezolizumab, etoposide and cisplatin) than in the chemoimmunotherapy group (Fig. 4D), supporting the idea that the YAP1 inhibitor enhanced the pro-apoptotic effects of chemoimmunotherapy. We also evaluated the effects of YAP1 inhibitor treatment combined with chemoimmunotherapy on colony formation and cancer stemness by a colony‐forming assay and flow cytometry (Figure S4). As shown in Figures S4A, B, in the DMS114 and H2286 cell lines, the combination of verteporfin and chemoimmunotherapy significantly decreased the number of colonies compared with chemoimmunotherapy alone (both *p* < 0.05). CD133 and CD44 are two commonly used markers for lung cancer stemness [56, 57]. In SCLC, tumor progression and drug resistance were closely related to cancer stemness [58]. The MFI values of CD133 and CD44 revealed that the addition of the YAP1 inhibitor to chemoimmunotherapy reduced cancer stemness in DMS114 (MFI of CD133: 32288 vs. 23123 ; MFI of CD44: 831000 vs. 185967) and H2286 (MFI of CD133: 43859 vs. 34348 ; MFI of CD44: 2610000 vs. 163408; Figures S4C, D) cells. In summary, targeting YAP1 effectively improved the efficacy of chemoimmunotherapy in the SCLC-Y subtype.

### Impact of YAP1 on PD-L1 expression and immune cells

**Fig. 5:**
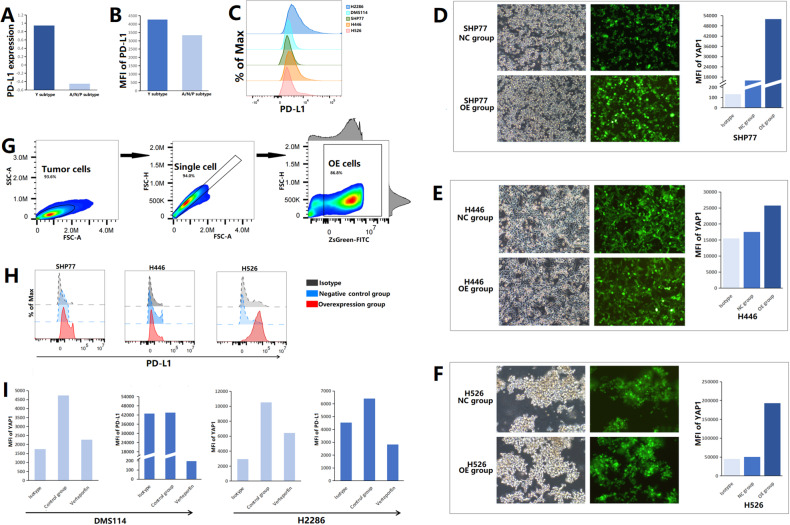
YAP1 regulated PD-L1 expression in small cell lung cancer (SCLC). **A** mRNA expression level of PD-L1 in different SCLC cell lines. **B**, **C** MFI of PD-L1 in different SCCL cell lines. **D, F** Microscopy of SHP77, H446, and H526 cell lines with stable overexpression of YAP1. Histograms depicted MFI of nuclear-localized YAP1 in SCLC-OE and NC cells. **G** Gating strategy of SCLC cells with stable overexpression of YAP1. **H** MFI of PD-L1 in OE and NC cells. **I** MFI of nuclear-localized YAP1 and PD-L1 in SCLC-Y cells after verteporfin exposure. MFI median fluorescence intensity, NC negative control, OE overexpression, PD-L1 program death-ligand 1, YAP1 yes-associated protein 1.

Several studies have noted the close relationship among YAP1, PD-L1, and immune cells [21, 59]. In our study, the SCLC-Y cell lines showed higher RNA and protein expression levels of PD-L1 than the SCLC-A, SCLC-N, and SCLC-P cell lines (Fig. 5A–C). To fully explore the correlation between YAP1 and PD-L1 expression, we generated SHP77, H446, and H526 cell lines with stable overexpression of YAP1 (Fig. 5D–F). Overexpression of nuclear-localized YAP1 in these cell lines was verified by flow cytometric analysis (Fig. 5D–G). Figure 5H reveals that the protein expression level of PD-L1 was increased with overexpression of YAP1 in SCLC cells. Similarly, inhibition of nuclear-localized YAP1 expression with verteporfin downregulated PD-L1 expression in DMS114 and H2286 cells (Fig. 5I). Overall, these results indicated that YAP1 could regulate PD-L1 expression in SCLC cells.

**Fig. 6:**
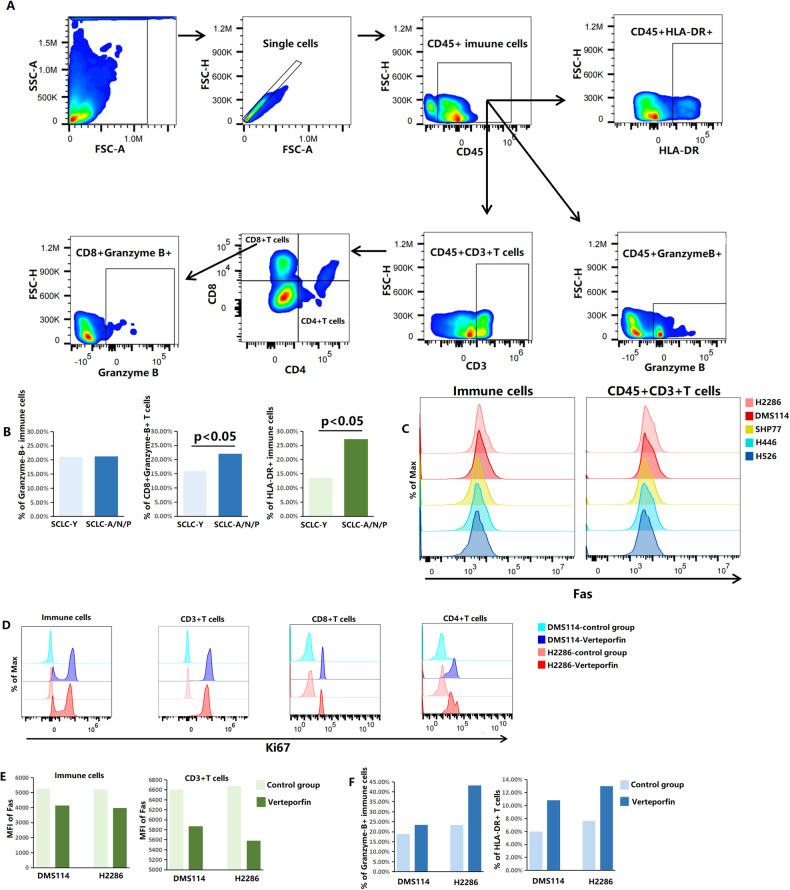
YAP1 modulated immune cell abundances, apoptosis, and functions in SCLC. **A** Gating strategy of different immune cells. **B** The proportions of different immune cells in the SCLC-Y group (DMS114 and H2286) and the SCLC-A/N/P group (SHP77, H446, and H526). **C** MFI of Fas in CD45+ immune cells and CD45 + CD3 + T cells in five coculture groups (tumor cells and PBMCs). **D** MFI of Ki67 in different immune cells in two coculture systems of SCLC-Y cells and PBMCs after verteporfin exposure. **E** MFI of Fas in CD45+ immune cells and CD45 + CD3 + T cells in two SCLC-Y and PBMCs coculture systems after verteporfin exposure. **F** The proportions of Granzyme-B+ immune cells and activated T cells (CD45 + CD3 + HLA-DR + ) in two coculture systems of SCLC-Y cells and PBMCs after verteporfin exposure. SCLC small cell lung cancer, MFI median fluorescence intensity, PBMCs peripheral blood mononuclear cells.

In the coculture system with SCLC cells and PBMCs, we explored the effects of YAP1 on immune cell infiltration and function by flow cytometric analysis (Fig. 6). In comparison with the coculture systems with SCLC-A/N/P cells (SHP77, H446, and H526) and PBMCs, the coculture systems containing tumor cells with high YAP1 expression (DMS114 and H2286) had significantly lower proportions of CD45 + CD3 + CD4-CD8+Granzyme-B + T cells (16% vs. 22%, *p* = 0.028) and activated immune cells (CD45 + HLA-DR + ; 13.59% vs. 27.23%, *p* = 0.044; Fig. 6B). Granzyme-B, a serine protease, reflects the function of cytotoxic lymphocytes in triggering target-cell apoptosis [60, 61]. HLA-DR is a reliable marker for assessing the activation and proliferation of T cells [62]. The above results suggested that high YAP1 expression might suppress cytotoxic activity of CD8 + T cells and activation of immune cells in SCLC. Fas plays an important role in inducing T-cell apoptosis [63]. High YAP1 expression was correlated with increased levels of Fas in both CD45+ immune cells (MFI: 5218–5260 vs. 3657–3953, *p* < 0.05) and CD45 + CD3 + T cells (MFI: 6602–6680 vs. 5038–5427, *p* < 0.05; Fig. 6C). Our findings indicated that increased expression of YAP1 might exert immunosuppressive effects on immune cells by promoting apoptosis.

To further confirm the impact of YAP1 on the proliferation, apoptosis, and cytotoxicity of immune cells, we inhibited the expression level of YAP1 with verteporfin in SCLC-Y cell lines (Fig. 6D–F). Ki67 is extensively used as a proliferation marker. In the DMS114 and H2286 coculture systems, increased proliferation of CD45+ immune cells, CD45 + CD3 + T cells, CD45 + CD3 + CD4-CD8 + T cells and CD45 + CD3 + CD4 + CD8- T cells was observed after inhibition of YAP1 expression with verteporfin (Fig. 6D). Moreover, there was a downward trend in lymphocytes apoptosis levels after verteporfin exposure in the DMS114 (MFI of Fas in immune cells: 5260 vs. 4143; in CD3 + T cells: 6602 vs. 5869) and H2286 (MFI of Fas in immune cells: 5218 vs. 3969; in CD3 + T cells: 6680 vs. 5578) coculture systems (Fig. 6E). YAP1 inhibition further promoted immune cell cytotoxicity and activation compared with those in the control group, with increased proportions of CD45+Granzyme-B+ immune cells (18.9% vs. 23.50% in the DMS114 coculture system; 23.40% vs. 43.20% in the H2286 coculture system) and CD45 + CD3 + HLA-DR + T cells (5.97% vs. 10.80% in the DMS114 coculture system; 7.59% vs. 13.00% in the H2286 coculture system; *p* = 0.036; Fig. 6F). These results highlighted that the application of verteporfin, a YAP1 inhibitor, could reverse immune suppression and promote immune cell function in the SCLC-Y subtype.

On the basis of the expression profile of each case, we explored TME cell infiltration characteristics mediated by YAP1 in the public cohort containing 81 clinical SCLC samples (Figure S5) [47]. By the ESTIMATE and CIBERSORT algorithms, the immune score, stromal score, tumor purity, and relative abundance of 22 types of cells in the TME were estimated. Figure S5A shows that high YAP1 expression group exhibited significantly higher immune scores (*p* = 5.2e-07) and higher stromal scores (p = 1e-11), and significantly lower tumor purity (*p* = 2.9e-09) than the low YAP1 expression group. The results for the above two groups obtained via the CIBERSORT algorithm and are visualized in Figure S5B–D. The correlation of each type of TME-infiltrating cell in the high YAP1 expression group was slightly stronger than that in the low YAP1 expression group (Figure S5C). Comparison between the two groups revealed significant component differences in 7 kinds of immune cells: memory B cells (*p* = 0.042), plasma cells (*p* = 0.015), resting dendritic cells (*p* < 0.001), resting mast cells (*p* = 0.02), monocytes (*p* = 0.032), resting memory CD4 + T cells (*p* < 0.001), and follicular helper T cells (*p* = 0.007). Overall, both our results and bioinformatics analysis results supported the idea that YAP1 might extensively modulate the abundance, apoptosis, activation, and cytotoxic function of immune cells in SCLC.

We also preliminarily investigated the changes in the tumor immune microenvironment after treatment with YAP1 inhibitor and chemoimmunotherapy (Figure S6). Notably, when compared with the chemoimmunotherapy regimen, the combination regimen (verteporfin plus chemoimmunotherapy) promoted the proliferation of T cells (Figure S6A) and inhibited apoptosis in T cells (MFI of Fas: 5385 vs. 5406 in the DMS114 coculture system; 4308 vs. 5755 in the H2286 coculture system; Figure S6B). The addition of the YAP1 inhibitor also promoted the activation and cytotoxic function of immune cells (Figure S6C, D). In the DMS114 cell coculture system, the percentage of CD45 + CD3 + HLA-DR + T cells increased from 9.29% in the chemoimmunotherapy group to 13.60% in the combination group (Figure S6C). In the H2286 cell coculture system, the abundance of activated T cells was more than two-fold higher in the verteporfin plus chemoimmunotherapy group than in the chemoimmunotherapy group (7.89% vs. 3.03%; Figure S6C). Figure S6D reveals that the proportion of CD45+Granzyme-B+ immune cells was increased to a certain degree by increasing verteporfin (25.7% vs. 18.1% in the DMS114 coculture system; 27.8% vs. 17.7% in the H2286 coculture system). Altogether, these results might explain the effectiveness of combination treatment with a YAP1 inhibitor and chemoimmunotherapy in the SCLC-Y subtype.

### Functional profiling of YAP1 in SCLC

To fully explore signal transduction pathways regulated by YAP1, we conducted gene function analysis (Figure S7). A total of 4913 DEGs were found between the high YAP1 expression group and the low YAP1 expression group, among which 39.5% (1940/4913) were upregulated in the high YAP1 expression group (Figure S7A). In addition, these DEGs were significantly enriched in immune-related pathways (Figures S7B, C), such as T-cell activation (GO:0042110, *p* = 2.2E-09), neutrophil activation (GO:0042119, *p* = 2.56E-35), cytokine-cytokine receptor interaction (hsa04060, *p* = 4.43E-17), chemokine signaling pathway (hsa04062, *p* = 1.19E-11), and TNF signaling pathway (hsa04668, *p* = 4.72E-12), etc. Figure S7D shows the top six YAP1-related gene sets with |ES | > 0.70 and FDR < 0.25: interferon alpha response, IL6-JAK-STAT3 signaling, interferon gamma response, allograft rejection, epithelial-mesenchymal-transition (EMT), and inflammatory response. The Jaccard indices were in the 0–0.15 range (Figure S7E). There were some overlapping genes among the above pathways. The greatest degree of overlap was found for IL4R and IL6, both was which were in five of the six gene sets (Figure S7F).

## Discussion

**Fig. 7:**
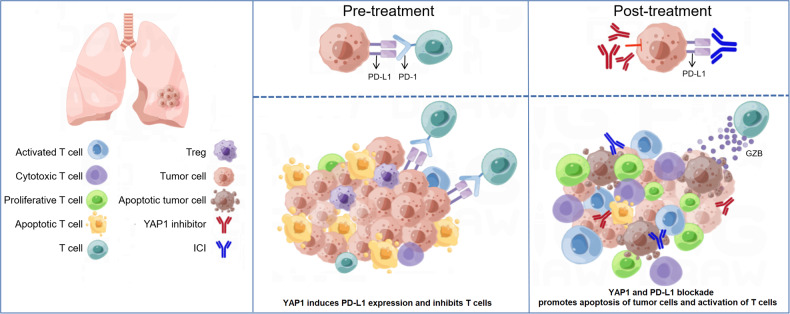
The molecular basis of YAP1 inhibitor in combination with immunotherapy in SCLC. High YAP1 expression induces PD-L1 expression, thus leading to the inhibition of T cells infiltration and function. The combinational treatment of YAP1 inhibitor and ICI significantly promotes apoptosis of tumor cells and activation of T cells. The addition of YAP1 inhibitor further enhances anti-tumor immunity. ICI immune checkpoint inhibitor, PD-1 program death-1, PD-L1 program death-ligand 1, SCLC small cell lung cancer, Treg regulatory T cells, YAP1 yes-associated protein 1.

With the proposal of the innovative SCLC molecular subtype classification [15], the immune landscapes and new options for combination therapy of the four subtypes remain to be fully investigated. In our study, we evaluated the expression patterns of the transcriptional regulators defining the four subtypes of SCLC and classified each clinical sample into one of the four subtypes by the NMF algorithm. Then, we outlined the immune profiles of and compared prognosis among the SCLC-A, SCLC-N, SCLC-P, and SCLC-Y subtypes. The worst prognosis of the SCLC-Y subtype was further validated in the public cohort. We found that the different molecular subtypes exhibited different sensitivities to immunotherapy. Specifically, the SCLC-Y subtype was sensitive to immunotherapy, consistent with previous research. In SCLC, YAP1 induced PD-L1 expression and inhibited immune cell activation and function (Fig. 7), which might explain the mechanism underlying the response of the SCLC-Y subtype to immunotherapy and provide a molecular basis for combining YAP1 inhibitor and PD-L1 inhibitor. By a CCK8 assay, colony formation assay, and flow cytometry, our team is the first to develop the strategy of co-targeting YAP1 and PD-L1 in SCLC, which dramatically boosted anti-tumor immunity and provided new ideas for combination therapy in patients with the SCLC-Y subtype. We also found that the addition of a YAP1 inhibitor to immunotherapy further enhanced anti-tumor activity and remodeled TME (Fig. 7), indicating the great potential of combining YAP1 and PD-L1 blockade in the clinical treatment of the SCLC-Y subtype, an approach that is expected to improve the prognosis of SCLC patients.

The proportions of these SCLC molecular subtypes were uneven. In a cohort with 81 SCLC samples and 54 cell lines, Rudin et al. [15] reported that the proportions of samples and cells with the SCLC-A, SCLC-N, SCLC-P, and SCLC-Y subtypes were 36%, 31%, 16%, and 17%, respectively. By means of IHC analysis, Qu et al. [64] identified 111 SCLC-A subtype, 8 SCLC-N subtype, 10 SCLC-P subtyp, and 4 SCLC-Y subtype tumors in a SCLC cohort with 146 primary tumors. In another SCLC cohort, the numbers of tumors with the SCLC-A, SCLC-N, SCLC-P, and SCLC-Y subtypes were 10 (16.9%), 3 (5.1%), 4 (6.8%), and 6 (10.2%), respectively [65]. Consistent with previous reports, in our study, the proportions of the SCLC-A and SCLC-N subtypes were the highest. By the analysis with the NMF algorithm, 32% of samples were classified into the SCLC-Y and SCLC-P subtypes in the local cohort, a percentage that was close to the reported percentage of 9.8%-33%. Early data indicated that most SCLC patients who underwent therapy exhibited the SCLC-N subtype, while treatment-naïve patients were more likely to be classified into the SCLC-A subtype [66]. In our cohort with 100 treatment-naïve SCLC patients, the proportion of patients with the SCLC-A subtype was higher than that of patients with the SCLC-N subtype (36% vs. 32%). In the primary cohort, a significant difference in RFS was observed among the 4 subtype groups, with the SCLC-Y subtype group exhibiting the least favorable prognosis. Similar results were obtained by bioinformatics analysis in the public SCLC cohort. A prior study underlined the poor prognosis of patients with the SCLC-Y subtype [15], an observation that was consistent with our findings and strongly supported our results. However, an opposite result was found in another published study [65]. Although significant differences were not found, Owonikoko et al. [65] revealed that the SCLC-Y subtype showed better OS and PFS outcomes than the other subtypes. There were two main reasons for the conflicting results. First, only 23 SCLC cases were successfully classified into one of the four molecular subtypes in the Owonikoko et al. [65] cohort. Second, the inconsistent findings might be ascribed to differences in clinical end points and the follow-up length. In studies with in vitro and vivo experiments, it was reported that activation of the Notch pathway promoted a shift from the neuroendocrine type to the non-neuroendocrine type and downregulated ASCL1 expression in SCLC [47, 67, 68]. Both the YAP1^+^ subtype and POU2F3^+^ subtype were characterized as non-neuroendocrine type [15]. Based on time-series single-cell RNA-sequencing data, a published study suggested that the SCLC molecular subtypes exhibited biological plasticity [69]. Their dynamic evolution was mediated by MYC [69]. Recently, the SCLC-I subtype, with high immune cell abundance and high expression of ICs, was identified in a SCLC clinical cohort [70].

An increased CD8^+^T cell count was found in the SCLC-P/Y (SCLC-P and SCLC-Y) group when compared with the SCLC-A/N (SCLC-A and SCLC-N) group [64]. By flow cytometry and multiplexed ion beam imaging, Chan et al. [59] showed that the SCLC-N subtype was characterized by an immune-cold phenotype with lower CD8^+^ immune cell infiltration and higher Treg proportion than the SCLC-A subtype. In this study, we preliminarily explored the infiltration of CD3^+^ T cells, CD4^+^ T cells, CD8^+^ T cells, and FOXP3^+^ Tregs in each SCLC molecular subtype. Similar results were found in our study, higher proportions of CD3+ and CD8 + T cells were observed in SCLC-P and SCLC-Y specimens than in SCLC-A and SCLC-N samples. Moreover, the SCLC-P subtype exhibited higher infiltration levels of CD4 + T cells and FOXP3+ Tregs than the other subtypes, while the SCLC-N subtype exhibited the lowest infiltration levels of CD3^+^, CD4^+^, and CD8^+^ T cells. Consistent with these findings, the infiltration level of FOXP3+ Tregs was higher in the SCLC-N subtype than in the SCLC-A subtype. In the present study, we also evaluated the differential expression levels of subtype marker genes among the four SCLC subtypes. Novel co-expression patterns of subtype marker genes and some ICs were proposed, that might provide new insights into the efficacy of combined immunotherapy for SCLC. As two key neuronal transcription factors, ASCL1 and NEUROD1, promoted the differentiation and maturation of pulmonary neuroendocrine cells [71–73]. In SCLC, POU2F3 was selectively distributed in one type of chemosensory cells termed tuft cells [24, 74]. As a main effector of the Hippo signaling pathway, YAP1 was mainly overexpressed in SCLC cells without RB mutation [75]. Contradictory findings were found in the co-expression pattern of ASCL1 and NEUROD1 in SCLC [15, 47, 76–81]. A low degree of ASCL1 and NEUROD1 co-expression was found in some studies [15, 47, 76–78], consistent with our results. In small cell lung carcinoma and lung adenocarcinoma, a significant relationship between YAP1 expression and either ASCL1 expression or POU2F3 expression was reported, while no significant association was found between YAP1 expression and NEUROD1 expression [15, 80].

YAP1 profoundly modulates cell migration, tumor growth, and cancer stemness by complex mechanisms, such as the Hippo pathway, EMT pathway, epigenetic modification, and regulation of immune cell infiltration [82–89]. In pancreatic cancer, inhibition of the Hippo/YAP1/c-Jun axis could suppress cancer stemness and overcome drug resistance [90]. The results of our study also indicated that the inhibition of YAP1 decreased stemness in SCLC cells. YAP1 had dual effects on the immune microenvironment. In melanoma, YAP1 played an important role in activating Tregs, thus facilitating the generation of an immunosuppressive TME [87]. YAP1 suppressed the differentiation of B cells and promoted B-cell dysfunction by BCR signaling pathway [89, 91]. In liver carcinoma, YAP1 promoted the infiltration of macrophages by upregulating the expression of monocyte chemoattractant protein-1 [92]. In colorectal cancer, M2 macrophage polarization was accelerated by YAP1, resulting in tumor formation [93]. Unexpectedly, through boosting the abundance of CD8 + T cells, the overexpression of YAP1 was found to inhibit the growth of melanoma cells, squamous cell carcinoma cells, and breast cancer cells [94]. In NSCLC and melanoma, the expression level of PD-L1 was regulated by YAP1 [95–98], while little was known about the role of YAP1 in SCLC. Our research might supplement the insufficient knowledge in this field and further prove the vital effects of YAP1 on PD-L1 expression and TME regulation. Overexpression of YAP1 also contributed to poor outcomes and drug resistance in non-SCLC (NSCLC), liver cancer, stomach cancer, breast cancer, and colorectal carcinoma, in addition to SCLC [99–103]. In SCLC, YAP1 was found to induce chemoresistance via CD74-related pathways and the notch pathway [54, 55, 75]. A hypothesis that the SCLC-Y subtype was sensitive to immunotherapy was proposed [15]. However, this hypothesis has not yet been confirmed. By a cell viability assay and apoptosis analysis, we confirmed that the SCLC-Y subtype could benefit more from immunotherapy than from chemotherapy. A similar trend was not found in the other SCLC subtypes. Then, we applied reliable cell proliferation marker (Ki67), apoptosis index (Fas), cytotoxic effector molecule (Granzyme-B), and activation indicator (HLA-DR) to evaluate the status of immune cells. We found that YAP1 promoted PD-L1 expression and inhibited T-cell functions in SCLC. Inhibition of YAP1 reversed immunosuppression in the SCLC-Y subtype. Targeting YAP1 and PD-L1 further reinforced the anti-tumor immunity. We investigated the transcriptome, functional profiles, and TME cell infiltration characteristics related to YAP1 in SCLC. We found that YAP1 expression was associated with high immune score, high stromal score, high tumor heterogeneity, and decreased infiltration of plasma cells and follicular helper T cells, indicating that remodeling the immune microenvironment might guarantee clinical benefits in SCLC patients with high YAP1 expression. Significant enrichment of T-cell activation signaling, cytokine binding, interleukin-related signaling, and chemokine signaling pathways were found in the high YAP1 expression group, suggesting that these might be the potential mechanisms of YAP1 in regulating the immune microenvironment. Together, these evidences highlight that YAP1 enhances immune evasion through inducing PD-L1 expression, remodeling the tumor immune microenvironment, and modulating immune-related signaling pathways. More extensive investigation of the complex mechanisms suggested by these findings is necessary.

There are still some limitations in our present research. First of all, clinical samples and data of the primary cohort were collected retrospectively. Secondly, we conducted the study and drew conclusions on the basis of two SCLC cohorts with relatively small sample sizes. More prospective studies with larger sample sizes are warranted. Moreover, the function of YAP1 is intimately tied to its subcellular localization and phosphorylation level. In endometrial cancer, Wang et al. found that nuclear-localized YAP enhanced cell proliferation [104]. The function of YAP1 was found to be inhibited by verteporfin through upregulation of the 14–3–3σ protein [104]. Thus, efforts to further explore the mechanisms linking changes in the subcellular localization and phosphorylation level of YAP1 to its function in SCLC are currently underway. In addition, we will carry out multi-omics experiments and clinical trials to further confirm our present findings and evaluate the safety and efficacy of YAP1 inhibitor treatment in combination with immunotherapy in SCLC.

## Conclusion

In conclusion, we analyzed the immune characteristics of each SCLC molecular subtype at the proteome level. In SCLC, YAP1 mediates immune escape by inducing PD-L1 expression and T-cell dysfunction. We found that the addition of a YAP1 inhibitor to immunotherapy further enhanced anti-tumor activity and immune cell function, indicating the great potential of combined YAP1 and PD-L1 blockade in the clinical treatment of patients with the SCLC-Y subtype, an approach that is expected to improve the prognosis of SCLC patients.

### Supplementary information

Figure S1

Figure S2

Figure S3

Figure S4

Figure S5

Figure S6

Figure S7

Table S1

Table S2

Reproducibility checklist

## Data Availability

Researches who provide a methodologically sound proposal for use of the data could sent emails to the address below to obtain the shared data: 1206339230@qq.com.
