# Supplementary figures and images for: YAP1 expression is associated with survival and immunosuppression in small cell lung cancer
Source: Cell Death Dis. 2023 Sep 26;14(9):636. doi: 10.1038/s41419-023-06053-y (PMC10522695; doi:10.1038/s41419-023-06053-y)

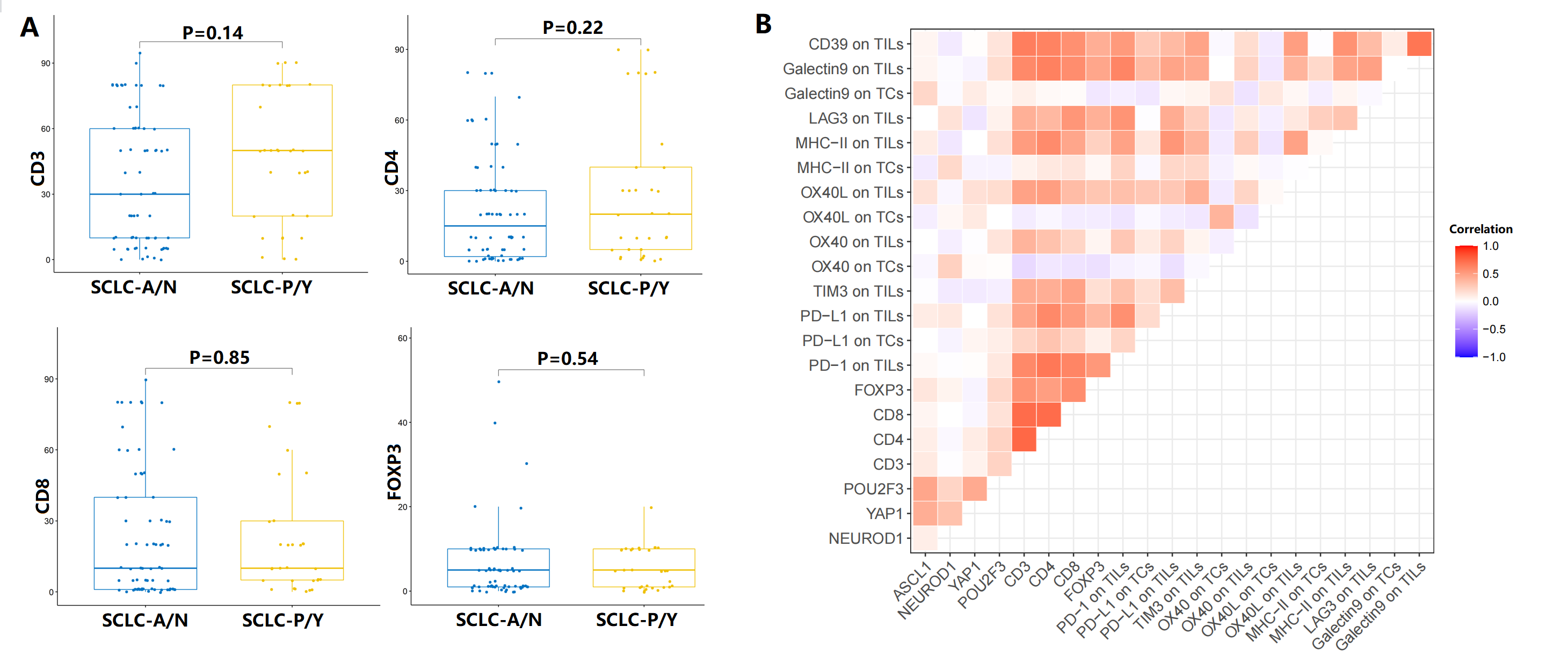

Supplement: Supplementary file 1 — Figure S1 [file 41419_2023_6053_MOESM1_ESM.png]

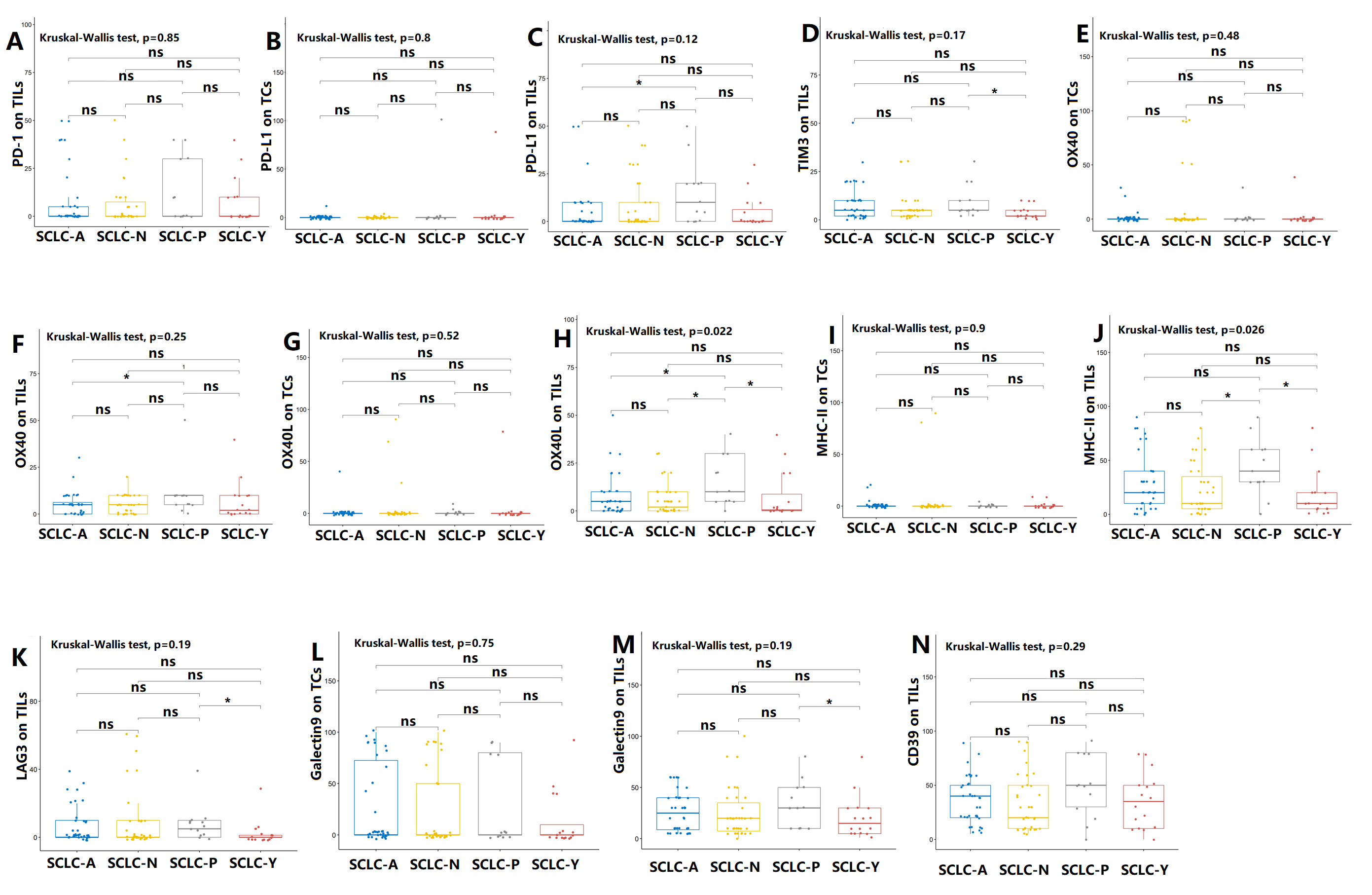

Supplement: Supplementary file 2 — Figure S2 [file 41419_2023_6053_MOESM2_ESM.png]

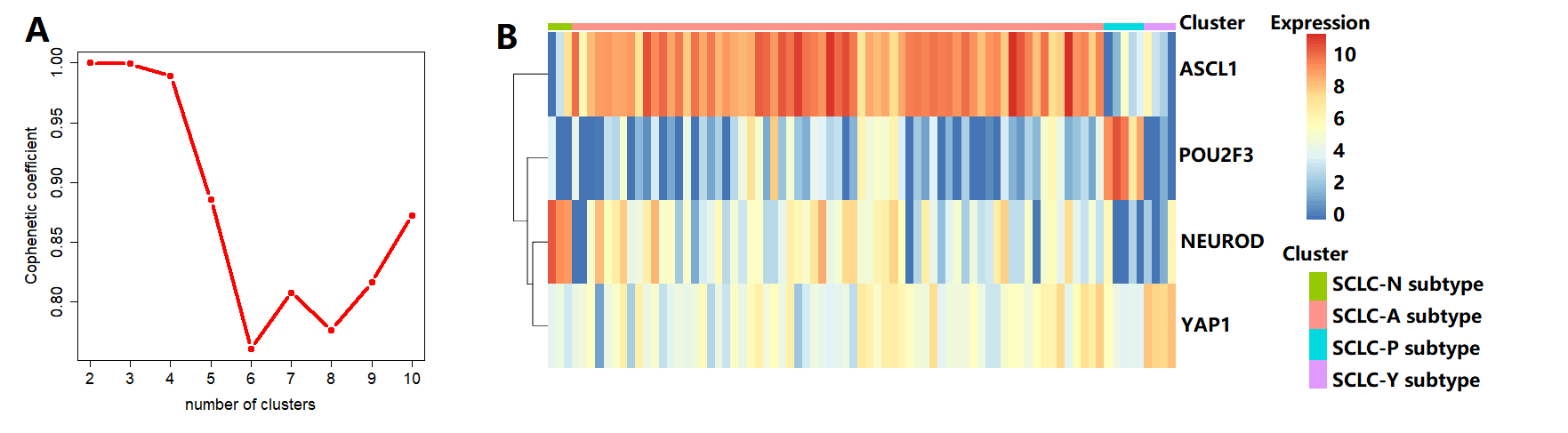

Supplement: Supplementary file 3 — Figure S3 [file 41419_2023_6053_MOESM3_ESM.png]

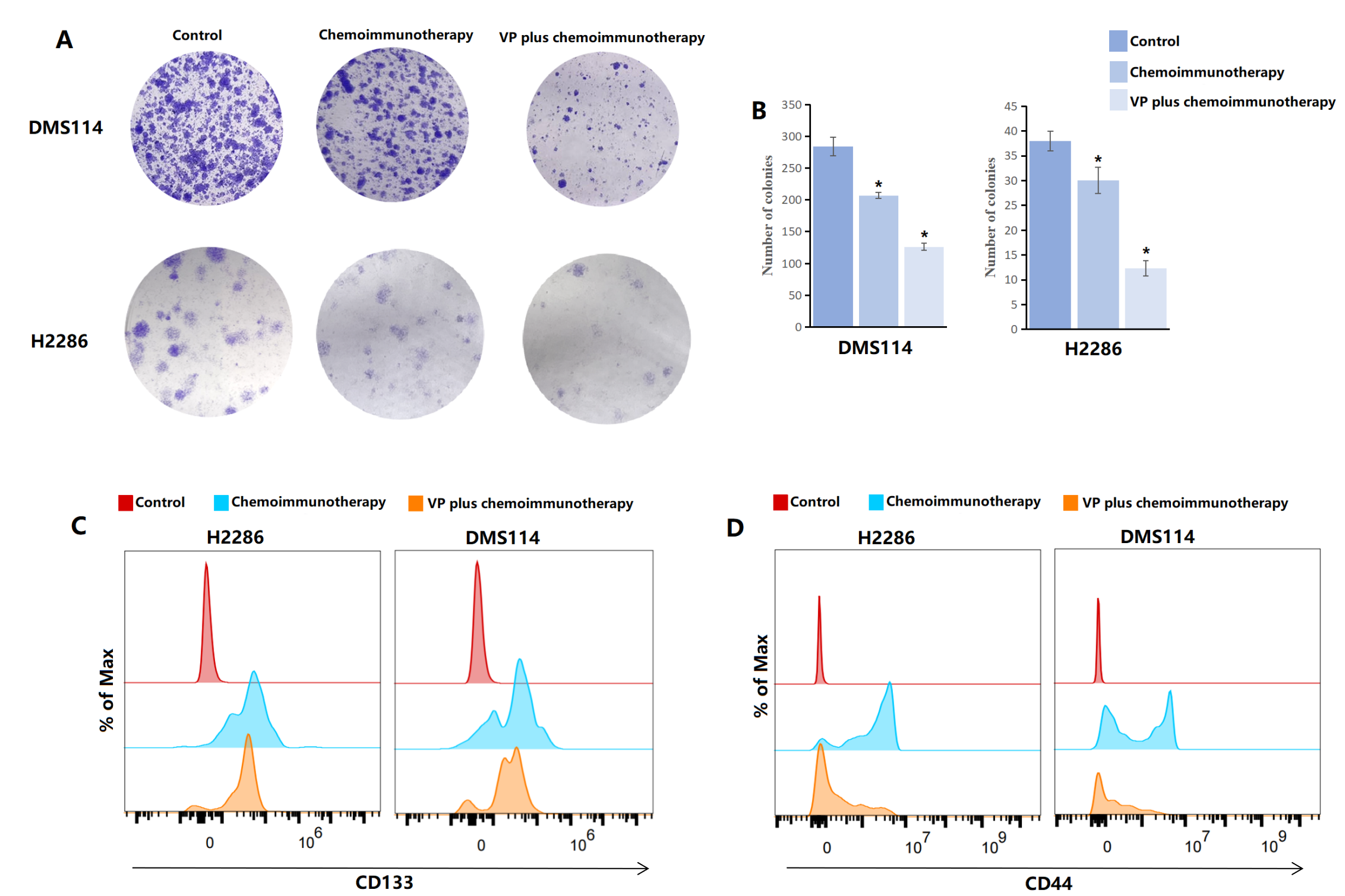

Supplement: Supplementary file 4 — Figure S4 [file 41419_2023_6053_MOESM4_ESM.png]

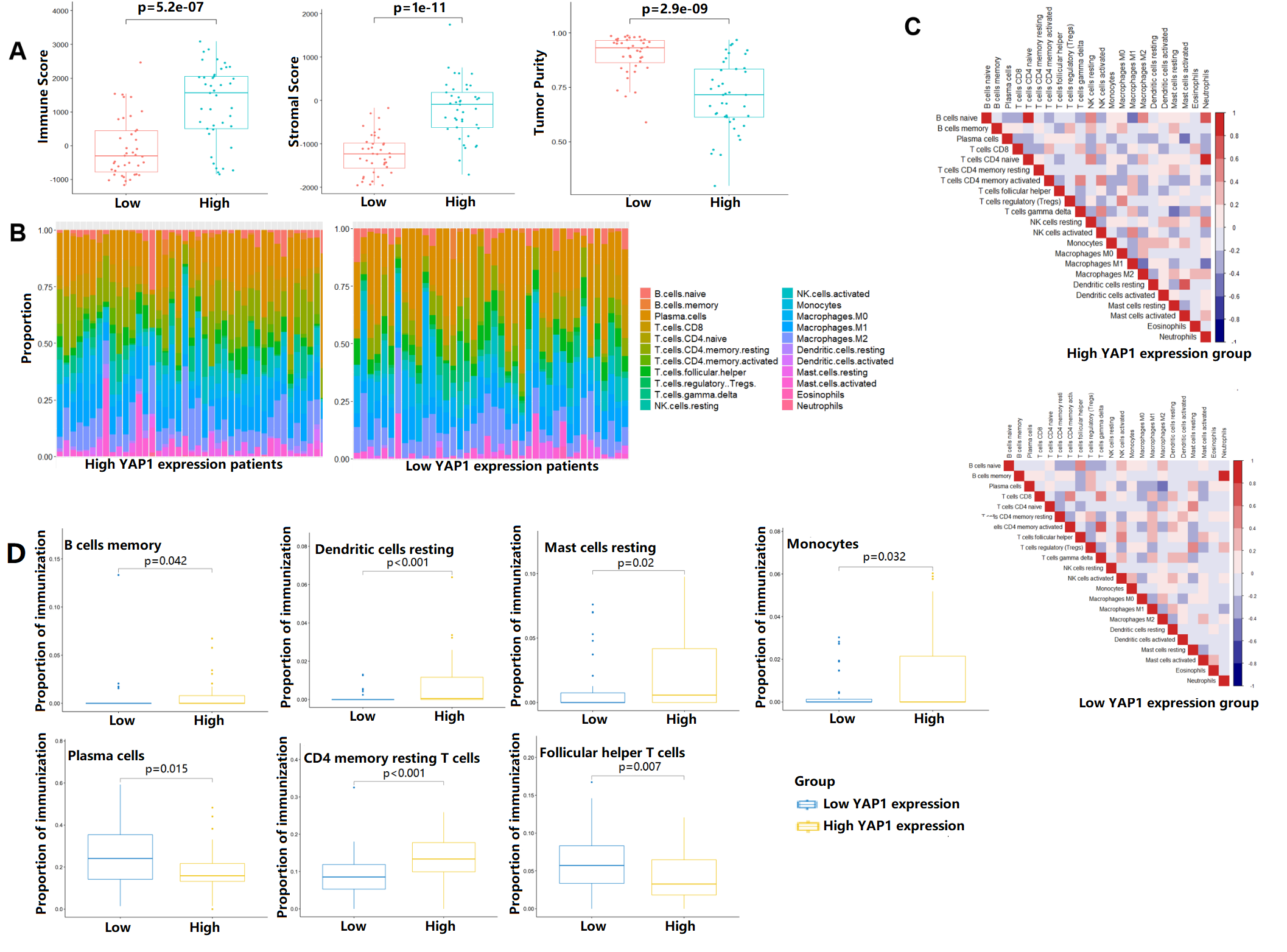

Supplement: Supplementary file 5 — Figure S5 [file 41419_2023_6053_MOESM5_ESM.png]

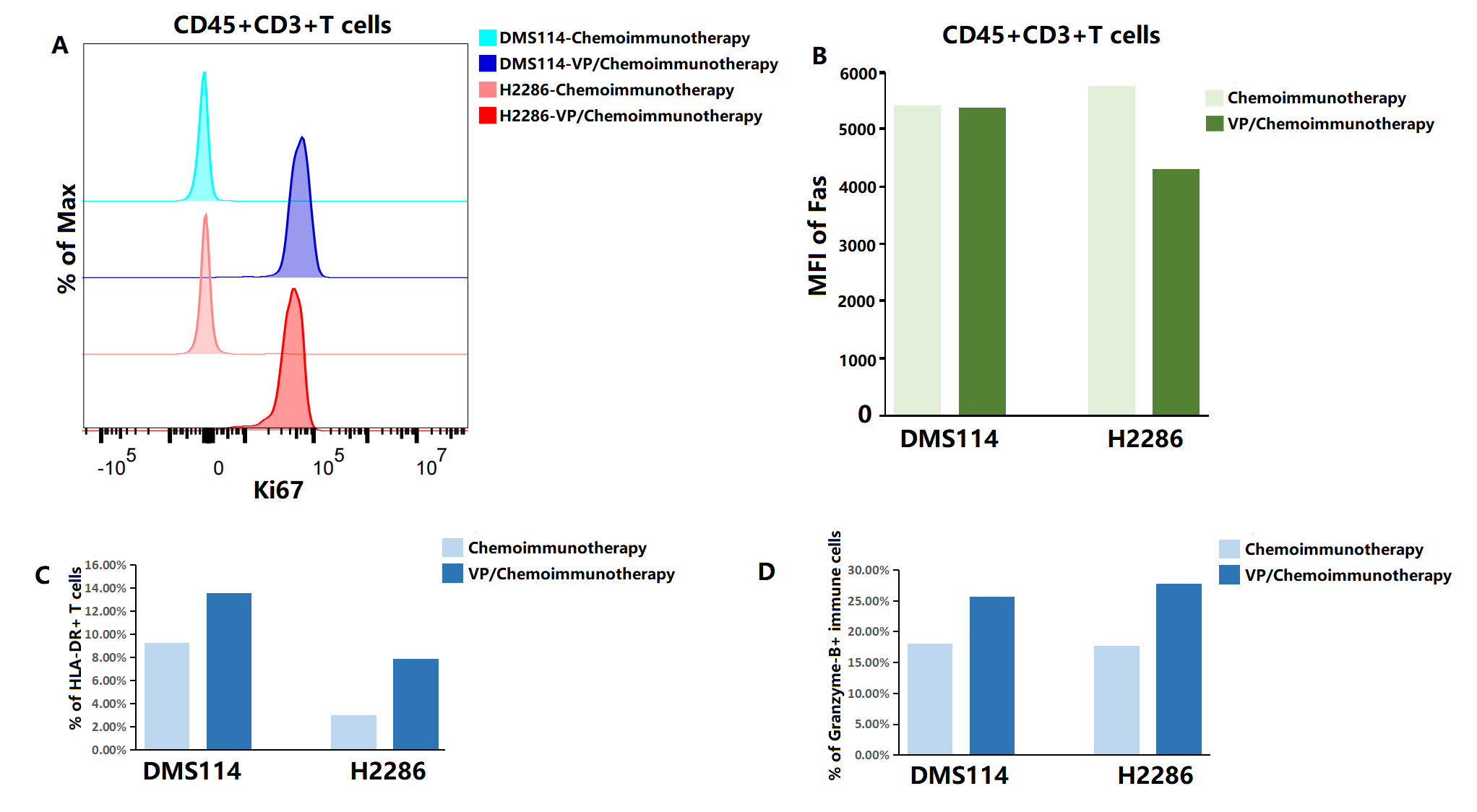

Supplement: Supplementary file 6 — Figure S6 [file 41419_2023_6053_MOESM6_ESM.png]

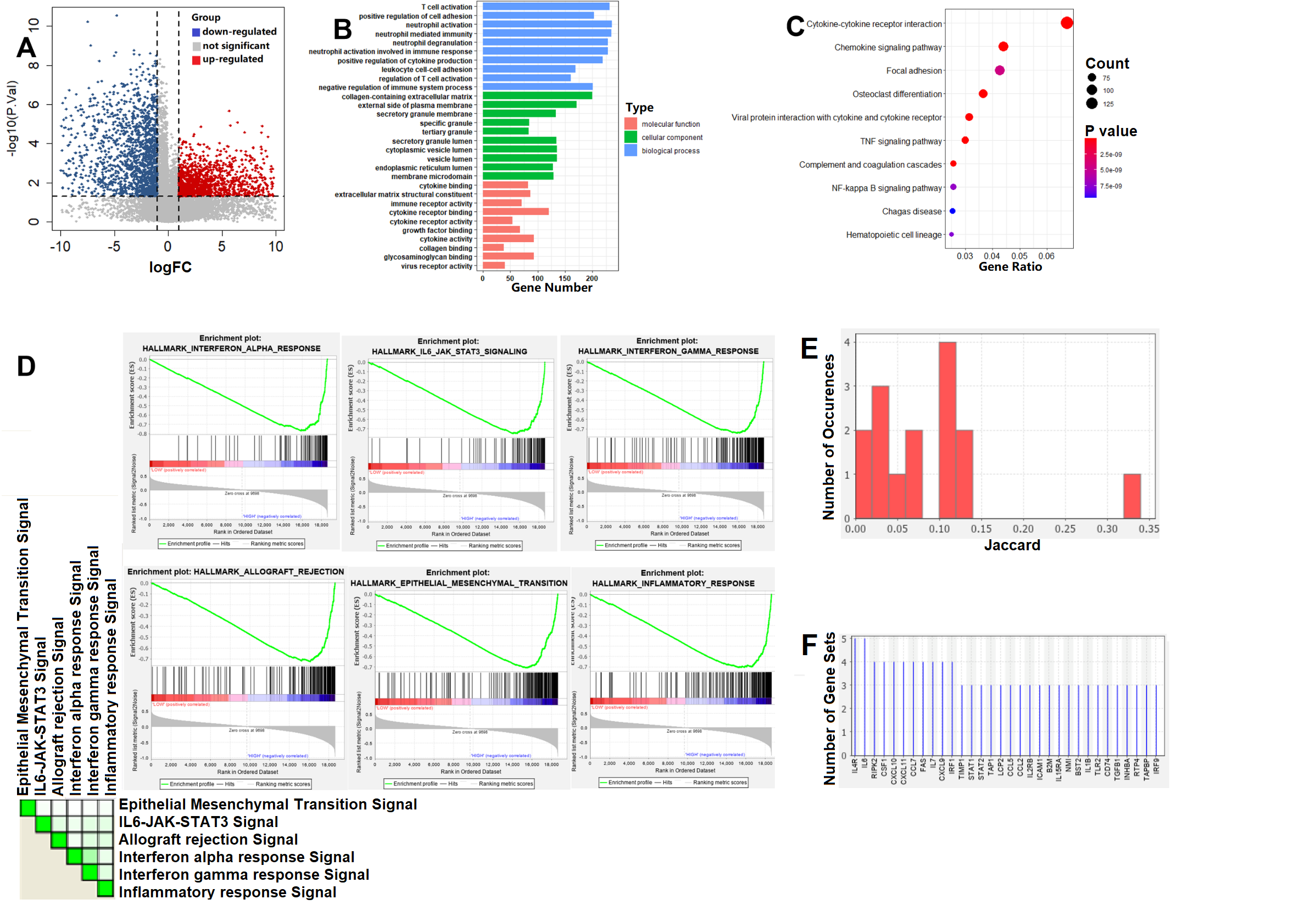

Supplement: Supplementary file 7 — Figure S7 [file 41419_2023_6053_MOESM7_ESM.png]
