# Supplementary material for: YAP1 expression is associated with survival and immunosuppression in small cell lung cancer
Source: Cell Death Dis. 2023 Sep 26;14(9):636. doi: 10.1038/s41419-023-06053-y (PMC10522695; doi:10.1038/s41419-023-06053-y)
Supplement: Supplementary file 8 — Table S1 [file 41419_2023_6053_MOESM8_ESM.docx]

**Table S1. Clinicopathological characteristics of the primary cohort (n=100)**

| **Variables** | **No.** | **Variables** | **No.** |
| --- | --- | --- | --- |
| Sex |  | T stage |  |
| Female | 18 | T1-2 | 86 |
| Male | 82 | T3-4 | 14 |
| Age, median, years | 63.5 | N stage |  |
| <65 | 52 | N0 | 44 |
| ≥65 | 48 | N1-3 | 56 |
| SCLC TNM staging^#^ |  | Postoperative treatment |  |
| I-II | 60 | No | 35 |
| III | 40 | Yes | 65 |

**Abbreviation:** N, lymph node; SCLC, small cell lung cancer; T, tumor; TNM, tumor-node-metastasis; ^#^SCLC operative staging.
