# Supplementary material for: YAP1 expression is associated with survival and immunosuppression in small cell lung cancer
Source: Cell Death Dis. 2023 Sep 26;14(9):636. doi: 10.1038/s41419-023-06053-y (PMC10522695; doi:10.1038/s41419-023-06053-y)
Supplement: Supplementary file 9 — Table S2 [file 41419_2023_6053_MOESM9_ESM.docx]

**Table S2. Clinicopathological characteristics of the public cohort (n=132)**

| **Variables** | **No.** | **Variables** | **No.** |
| --- | --- | --- | --- |
| Sex |  | T stage |  |
| Female | 28 | T1-2 | 38 |
| Male | 104 | T3-4 | 16 |
| Age |  | NA | 78 |
| <65 | 80 | N stage |  |
| ≥65 | 52 | N0 | 45 |
| SCLC molecular subtype |  | N1-3 | 78 |
| SCLC-A | 103 | NA | 9 |
| SCLC-N | 13 | SCLC TNM staging |  |
| SCLC-P | 11 | I-II | 74 |
| SCLC-Y | 5 | III-IV | 58 |

**Abbreviation:** N, lymph node; NA, not available; SCLC, small cell lung cancer; T, tumor; TNM, tumor-node-metastasis.
